# Differential expression of type X collagen in a mechanically active 3-D chondrocyte culture system: a quantitative study

**DOI:** 10.1186/1749-799X-1-15

**Published:** 2006-12-06

**Authors:** Xu Yang, Peter S Vezeridis, Brian Nicholas, Joseph J Crisco, Douglas C Moore, Qian Chen

**Affiliations:** 1 Orthopaedic Research Laboratories, Department of Orthopaedics, Brown Medical School/Rhode Island Hospital, Providence, RI 02903, USA

## Abstract

**Objective:**

Mechanical loading of cartilage influences chondrocyte metabolism and gene expression. The gene encoding type X collagen is expressed specifically by hypertrophic chondrocytes and up regulated during osteoarthritis. In this study we tested the hypothesis that the mechanical microenvironment resulting from higher levels of local strain in a three dimensional cell culture construct would lead to an increase in the expression of type X collagen mRNA by chondrocytes in those areas.

**Methods:**

Hypertrophic chondrocytes were isolated from embryonic chick sterna and seeded onto rectangular Gelfoam sponges. Seeded sponges were subjected to various levels of cyclic uniaxial tensile strains at 1 Hz with the computer-controlled Bio-Stretch system. Strain distribution across the sponge was quantified by digital image analysis. After mechanical loading, sponges were cut and the end and center regions were separated according to construct strain distribution. Total RNA was extracted from the cells harvested from these regions, and real-time quantitative RT-PCR was performed to quantify mRNA levels for type X collagen and a housing-keeping gene 18S RNA.

**Results:**

Chondrocytes distributed in high (9%) local strain areas produced more than two times type X collagen mRNA compared to the those under no load conditions, while chondrocytes located in low (2.5%) local strain areas had no appreciable difference in type X collagen mRNA production in comparison to non-loaded samples. Increasing local strains above 2.5%, either in the center or end regions of the sponge, resulted in increased expression of Col X mRNA by chondrocytes in that region.

**Conclusion:**

These findings suggest that the threshold of chondrocyte sensitivity to inducing type X collagen mRNA production is more than 2.5% local strain, and that increased local strains above the threshold results in an increase of Col X mRNA expression. Such quantitative analysis has important implications for our understanding of mechanosensitivity of cartilage and mechanical regulation of chondrocyte gene expression.

## Background

Cartilage in human joints is subjected to various loads that regulate chondrocyte metabolism and cartilage extracellular matrix protein composition. The mechanical stress placed on cartilage *in vivo *plays an important role in the regulation of chondrocyte proliferation, differentiation, and hypertrophy. One of the ways in which this regulation occurs is through complex control of chondrocyte gene expression. Mechanical loading of cartilage is sensed by chondrocytes embedded within extracellular matrix. Mechanical signals then activate mechanotransduction pathways to alter gene expression [1-3]. These chondrocyte mechanoregulatory pathways are hypothesized to involve several levels of signaling, including transduction through ion channels [2], activation of transcription factors [4], and alteration of microtubules in the cytoskeleton [5].

Previous study using the Bio-Stretch culture system has demonstrated that chondrocytes subjected to tensile strain maintain their chondrocyte phenotype [2]. These cells are stimulated first to proliferate and then to mature and hypertrophy by the cyclic uniaxial tensile strain induced by the device [2]. We identified the type X collagen gene as one of the mechanosensitive genes in cartilage [2]. Type X collagen is a marker for hypertrophic cartilage since its mRNA is greatly up regulated in hypertrophic chondrocytes. Interestingly, type X collagen mRNA is induced in articular chondrocytes during osteoarthritic pathogenesis [6-9]. It is not clear how type X collagen mRNA expression is stimulated only in a specific part of cartilage, e.g., the hypertrophic region and/or the osteoarthritic lesion. Elucidation of the differential expression of type X collagen regulated by mechanical loading will provide a clearer understanding of the mechanoregulatory pathways involved in normal and pathogenic cartilage processes.

Our previous study has shown that type X collagen mRNA is significantly up regulated in response to 5% overall matrix deformation at 1 Hz in a 3-D chondrocyte culture system after 48 hours cyclic loading [2]. The specific loading strain and frequency were chosen because they stimulate the proliferation and differentiation of growth plate chondrocytes [2]. In the present study, we test the hypothesis that various local strains in different regions of the 3D scaffold result in different levels of type X collagen mRNA expression by chondrocytes in those areas.

## Methods

### Chondrocyte isolation

Primary cultures of early hypertrophic chondrocytes were established from 17-day-old embryonic chick sterna as described previously [10,11]. Chondrocytes from the cephalic part of chick sterna were used in the examination of type X collagen mRNA levels. Briefly, sternal cartilage pieces were enzymatically dissociated using 0.1% trypsin (Sigma, St. Louis, MO, USA), 0.3% collagenase (Worthington, Freehold, NJ), and 0.1% type I testicular hyaluronidase (Sigma). After an incubation of 30 min at 37°C and 5% CO_2_, the media was replaced and the incubation was continued at 37°C for an additional 1 h. Chondrocytes were centrifuged and suspended at 5 × 10^6 ^cells/ml in Ham's F-12 medium (Life Technologies, Grand Island, NY, USA) containing 10% fetal bovine serum (HyClone, Logan, UT, USA). One hundred μl of cell suspension was added into each sponge.

### 3D chondrocyte culture

**Figure 1:**
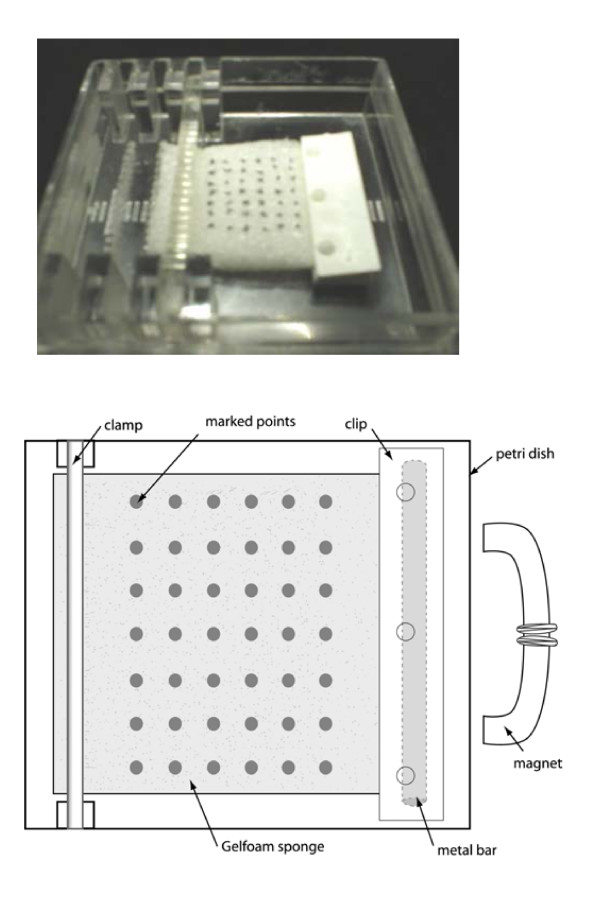
Photograph and line drawing of the Gelfoam sponge loaded in a square petri dish with a 6 by 7 grid of dots marked on surface. The stationary clamp edge is on left, and mobile plastic clip-metal bar assembly is on right.

Gelfoam sponges (Dupont, Delaware) were cut into rectangular pieces (2 cm × 2 cm), assembled in cell culture chambers, and seeded with chondrocytes as described previously [2]. The Bio-Stretch device (ICCT Technologies, Markham, ON, Canada) stretched the chondrocyte-seeded sponges at different overall strains (the extent of the deformation of the entire sponge) at 1 Hz with a duty cycle of 25%. Control chondrocyte-seeded sponges were maintained under identical test conditions with the exception that the sponges were not mechanically loaded. After 48 h of culture, sponges were washed once in HBSS, and 2 mm lengths from the fixed and free ends of each sponge (high strain) were cut and separated from the center area (low strain) (see Fig. 1 and 3). 2 mm lengths were examined since mechanical characterization of the Gelfoam sponge demonstrated that local strain decreased to a constant level of one-half overall strain 2 mm from each edge of the sponge. Chondrocytes were harvested by digestion of collagen sponge samples with 0.03% collagenase in HBSS for 20 min at 37°C. Cells were collected by centrifugation at 1000 rpm for 7 min and then resuspended in HBSS and counted with a hemacytometer (American Optical Corporation, Buffalo, NY, USA). Each of the four groups (non-stretch/stretch, center/ends) contained n = 5 samples.

**Figure 2:**
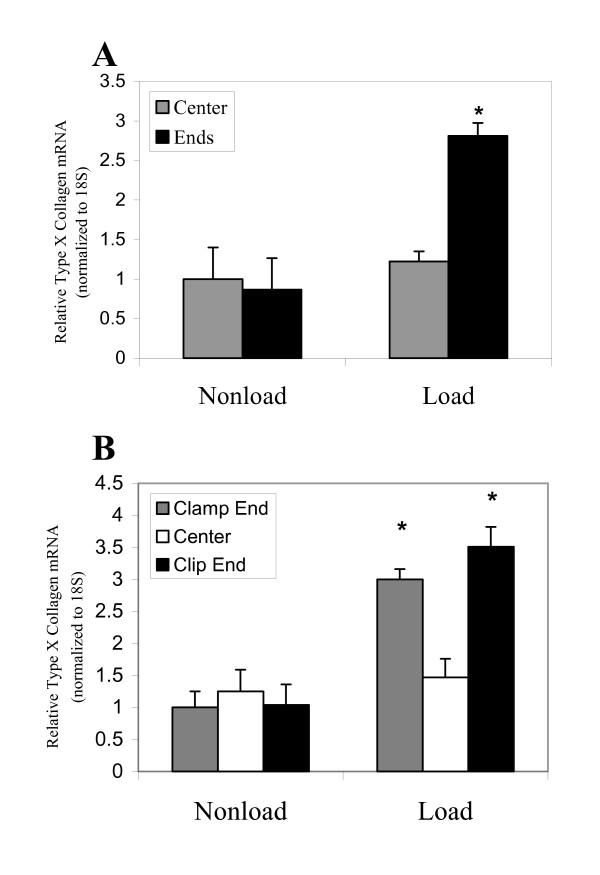
A. Chondrocytes from the ends of the sponge that experienced higher local strain had a statistically significant increase in type X collagen mRNA production in comparison to the corresponding region under no load conditions. (*: *p *< 0.05) n = 5. Type X collagen mRNA production was not significantly affected by loading in the center region of the sponge. B. Chondrocytes from both the clip end and the clamp end of the sponge had a statistically significant increase in type X collagen mRNA production in comparison to their corresponding regions under no load conditions. (*: *p *< 0.05) n = 5. Type X collagen mRNA expression levels in hypertrophic chondrocytes cultured in a sponge were subjected to 5% overall strain. ColX mRNA was quantified using real-time quantitative RT-PCR. The mRNA levels were normalized to 18S RNA levels, which served as the internal control.

**Figure 3:**
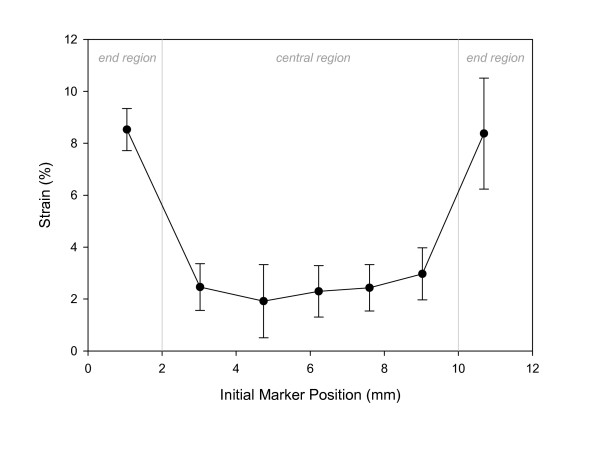
Distribution of surface strains in a typical sponge (4.3% overall strain in this example). The local strains in the central region were found to be dramatically lower than the strain in either end region. Strain values are reported as mean ± one standard deviation.

### Analysis of type X collagen mRNA levels

Total RNA was extracted from cells with RNeasy mini kits (Qiagen, Valencia, CA, USA). Quantification of the type X collagen mRNA was performed by real-time quantitative reverse transcriptase PCR (RT-PCR). 1 μg total RNA was used for each reverse transcriptase reaction in a reaction buffer containing 1 μl oligo(dT) and 1 μl 10 mM dNTP Mix (Invitrogen, Carlsbad, CA, USA). Real-time quantitative PCR amplification was performed using SYBR Green I (Finnzymes, Keilaranta, Finland) with DNA Engine Opticon 2 Continuous Fluorescence Detection System (MJ Research, Waltham, MA, USA). Primers used in amplification of type X collagen mRNA are shown in Table 1. Type X collagen mRNA levels were normalized to housekeeping gene 18S RNA levels. Since the level of 18S RNA is constant in all the cells, the normalized value reflected the relative level of type X collagen mRNA in each cell regardless of the cell number. Calculation of the type X collagen mRNA values was performed as previously described [2]. The 18S RNA was amplified at the same time and used as an internal control. The cycle threshold (Ct) values for 18S RNA and that of samples were measured and calculated by computer software (PE ABI). Relative transcript levels were calculated as x = 2^-ΔΔCt^, in which ΔΔCt = ΔE – ΔC, and ΔE = Ct_exp_-Ct_18s_; ΔC = Ct_ctl_-Ct_18s_.

**Table 1:** Oligonucleotide primer sequences used for real-time quantification RT-PCR detection of type X collagen mRNA

**Gene**	**Primer**	**Sequence**
Type X collagen	Forward	5'-AGTGCTGTCATTGATCTCATGGA-3'
	Reverse	5'-TCAGAGGAATAGAGACCATTGGATT-3'
18S RNA	Forward	5'-CGGCTACCACATCCAAGGAA-3'
	Reverse	5'-GCTGGAATTACCGCGGCT-3'

### Western blot analysis

Western blot analysis was performed with collected cell lysates from cell culture. Cell lysates were extracted using 4 M urea, 50 mM Tris at pH 7.5. For non-reducing condition, collected samples were mixed with standard 2× SDS gel-loading buffer. For reducing conditions, the loading buffer contains 5% b-mercaptoethanol and 0.05 M DTT. Samples were boiled for 10 minutes before loaded onto 10% SDS-PAGE gels. After electrophoresis, proteins were transferred onto Immobilon-PVDF membrane (Millipore Corp., Bedford, MA, USA) in 25 mM Tris, 192 mM glycine, and 15 % methanol. The membranes were blocked in 2% bovine serum albumin fraction V (Sigma Co., St. Louis, MO, USA) in PBS for 30 minutes and then probed with antibodies. The primary antibodies used were a polyclonal antibody against Col X [10], and a monoclonal antibody against β-actin. Horseradish peroxidase conjugated goat anti-mouse or goat anti-rabbit IgG (H+L) (Bio-Rad Laboratories, Melville, NY, USA), diluted 1:3,000, was used as a secondary antibody. Visualization of immunoreactive proteins was achieved using the ECL Western blotting detection reagents (Amersham Corp., Heights, IL, USA) and exposing the membrane to Kodak X-Omat AR film. Molecular weights of the immunoreactive proteins were determined against two different sets of protein marker ladders.

### Quantification of strain distribution across the sponge

Strain distribution was determined for collagen Gelfoam sponges (n = 4) loaded in the culture dish of the Bio-Stretch electromagnetic system (ICCT Technologies, Markham, ON, Canada). Gelfoam sponge (Upjohn, Kalamazoo, MI, USA) was cut into rectangular pieces (20 mm × 20 mm × 6 mm). A-plastic clip assembly with an imbedded metal bar was attached to one end of the sponge and the other end of the sponge was fixed to the culture dish with a plastic clamp leaving approximately a 12 mm length of exposed sponge. Using a fine tipped permanent marker, a 6 by 7 grid of dots was placed on each sponge to provide marker points for measurement of sponge strain distribution (Fig. 1). The sponge was then pre-soaked with Hanks' Balanced Salt Solution (HBSS, GIBCO, Grand Island, NY) overnight at 37°C and 5% CO_2_.

Sponges were deformed using power settings on the Bio-Stretch system of 20%, 30%, 40%, 50%, 60%, and 70%. Digital images of each sponge were captured in the unstretched and maximally stretched state at each power setting in 16-bit gray-scale at 16× magnification using a Polaroid DMC2 digital microscope camera (Polaroid, Wayland, MA, USA) connected to a Leica M26 stereomicroscope (Leica, Bannockburn, IL, USA). Scion Image software (Scion, Frederick, MD, USA) was used to analyze the sponge images. Using this software, each image was thresholded to assign x- and y-coordinate values to the centroid of each marker point. The x- and y-coordinate values of points along the clamp edge and clip edge were also recorded. The x-direction was defined in the direction of the principal tensile load and the y-direction was in the perpendicular direction. The local strain was calculated as a change in length between unstretched and stretched positions as a percent of the unstretched state. Strain values were calculated for all combinations of adjacent marker points. The strain in the transverse direction (y direction) was zero at both ends because the sponge was clamped at each end and ranged from undetectable values at the lower power to very small values at maximum power. Thus all strain values reported here in are those in the x-direction. Strain values are reported with respect to their initial unstretched position on the sponge and are the averages of the strain values for that specific column (y-direction) of marker points.

### Statistical analysis

Two-tailed t-tests were used to compare type X collagen mRNA levels from mechanically loaded chondrocytes in the Gelfoam sponge to those in the corresponding region under non-load conditions. Col X mRNA levels from chondrocytes in the center or end regions of the sponge in response to different strains were analyzed by one-way ANOVA with Dunnett Multiple Comparison post-hoc test. For these calculations, *p *< 0.05 was considered to be statistically significant.

## Results

### Type X collagen mRNA expression in response to 5% overall strain

We have shown previously that hypertrophic chondrocytes significantly increased their Col X mRNA production in response to 5% overall strain following 48 h cyclic uniaxial mechanical loading [2]. However, we found that type X collagen mRNA levels were not up regulated by chondrocytes in the center region of sponges, defined as the central region 2 mm from each end, in response to cyclic mechanical loading (Figure 2). In contrast, hypertrophic chondrocytes from the 2 mm areas at the ends of the sponge (end region) produced more than 2 times of type X collagen mRNA compared to those in the end region of non-loaded sponge (Figure 2A). Chondrocytes from both ends of the sponge produced significantly higher levels of Col X mRNA under loading conditions than the corresponding regions under non-load conditions (Figure 2B). Therefore, the increase of Col X mRNA level in response to 5% overall strain was attributed to the chondrocytes residing in the end regions, but not those in the central region of the sponge.

### Strain distribution across the collagen sponge

**Figure 4:**
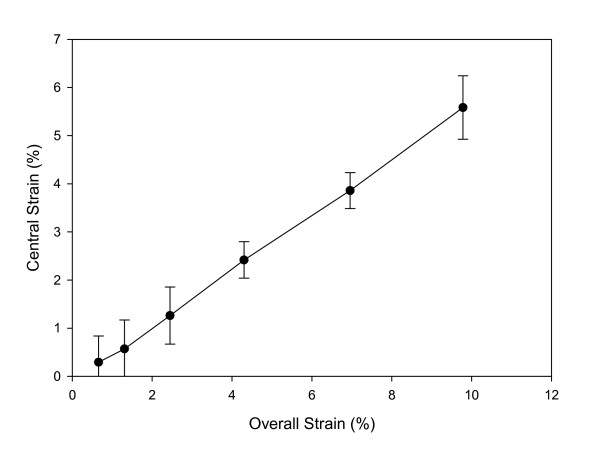
Relationship between strains in the central region versus overall strains. The strain values in the central region were approximately 1/2 (0.5 ± 0.07; n = 4) of the overall strain across a wide range of overall strain values generated by various power settings on the Bio-Stretch System. Each point in the graph represents a different power level tested.

Quantification of the surface strains of a Gelfoam sponge indicated that mechanical property was different in the end region vs. the central region of collagen scaffold. Tensile loading of the sponge by the Bio-Stretch system resulted in a highly non-uniform strain distribution – the strain in the end region was much higher than the strain in the central region (Figure 3). As a result, 5% overall strain caused 2.5% local strain in the central region and 9% local strain in the end region of a sponge. However, the strain in the central region of the sponge was nearly constant. This constant strain in the central region was consistently 1/2 of the overall strain values across a wide range of overall strain values tested. Specifically, for the six groups of overall strain values tested, the ratio of central strain to overall strain was 0.497 ± 0.067 (Figure 4).

### Type X collagen expression in response to different overall strains

**Figure 5:**
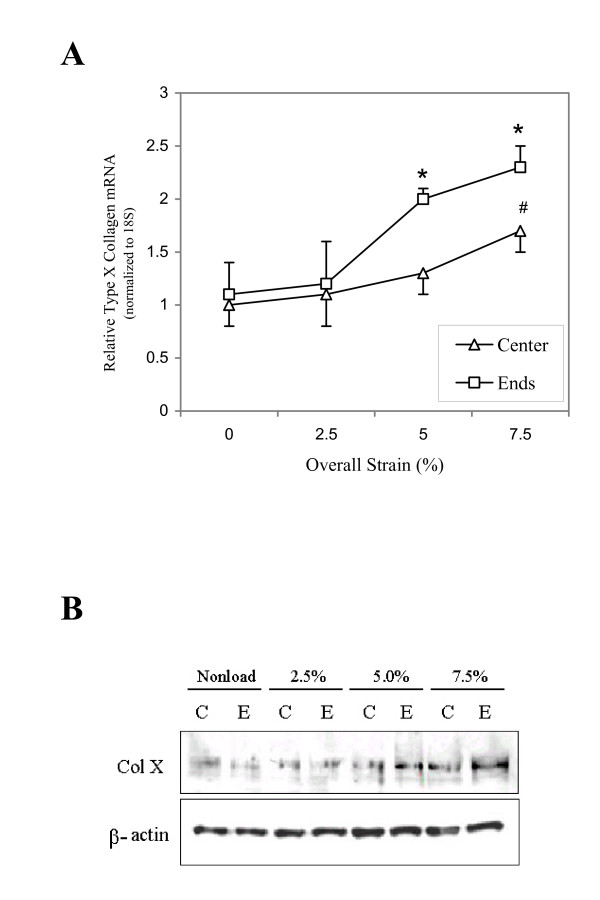
A. Type X collagen mRNA expression levels in hypertrophic chondrocytes cultured in different sponges subjected to different overall strains. Quantifying ColX mRNA was performed using real-time quantitative RT-PCR. The mRNA levels were normalized to 18S RNA, which served as the internal control. Chondrocytes from the central region of sponges subjected to 7.5% overall strain (3.75% local strain) had a significant increase in type X collagen mRNA production compared to the central region of non-loaded (0% strain group) sponges (n = 3/group; #: *p *= 0.02). Chondrocytes from the end region of the sponges subjected to 5% or 7.5% overall strains had a significant increase in type X collagen mRNA production in comparison to the end region of non-loaded (0% strain group) sponge (n = 3/group; *: *p *< 0.01). B. Western blot analysis of type X collagen from hypertrophic chondrocytes cultured in different sponges subjected to different overall strains. β-actin was used as an internal control of a housekeeping protein. Note the increasing strains result in an increase of type X collagen protein level while the level of β-actin remains constant. C: the center region of sponge; and E: the end region of sponge. Data shown are representative of those from three independent experiments.

To determine whether type X collagen mRNA production was affected by the overall strain of a sponge, we quantified Col X mRNA levels from both central and end regions of the sponges subjected to different overall strains including 0% (non-load), 2.5%, 5%, and 7.5% (Figure 5A). For the central region, only the Col X mRNA value from the 7.5% overall strain group was significantly (p = 0.02) higher than that from the central region of non-loaded sponge (0% strain group). This indicated a local strain at 3.75% (half of the overall strain) is required for up regulation of Col X mRNA. For the end regions, samples from 5% and 7.5% overall strain groups, but not that from 2.5% overall strain group, had significantly (p < 0.01) higher Col X mRNA levels than that from the end region of non-loaded sample. Therefore, Col X mRNA production was increased with increasing local strains regardless of the region of sponge. We also quantified Col X protein production by chondrocytes in the center and end regions of the sponge subjected to different overall strains (Figure 5B). Western blot analysis indicated that Col X protein levels were up regulated in the samples from higher strain regions (5% End, 7.5% Center, and 7.5% End). Thus increasing overall strains results in an increase of Col X protein production.

## Discussion

This study tested the hypothesis that mechanical microenvironment resulting from higher magnitudes of local strain within a three-dimensional chondrocyte culture system leads to increased type X collagen mRNA expression by chondrocytes in those areas. This hypothesis was tested in two ways: 1) in a single sponge in response to different local strains, and 2) in different sponges in response to different overall strains. Data from both tests supported the conclusion that induction of Col X mRNA was resulted from an increasing local strain above a certain threshold.

First, taking advantage of the non-uniform strain distribution property of the sponge, we demonstrated that type X collagen mRNA expression in hypertrophic chondrocytes subjected to cyclic matrix deformation is dependent on differential local strains within the same sponge. Under identical culture conditions, chondrocytes in the region experiencing high local strain produced higher levels of type X collagen mRNA than those under non-loaded conditions, while there was no significant difference of Col X production between the region experienced low local strain and that under no strains. Interestingly, non-uniform strain distribution as described for the collagen sponge exists in articular cartilage, with the highest strain observed in the end zones of cartilage [12,13]. The system utilized in the present study exerts differential local strains within the collagen scaffold of implanted chondrocytes. This property is significant in that it allows for differential strains within a single cell culture chamber, thereby limiting variation in the cell culture environment of the chondrocytes. However, one precaution is the local strain values measured in the present study represent surface strains, because the strains on the interior of the sponge in the end region could not be determined. Furthermore, there is not necessarily a distinct transition from an area of high strain to an area of low strain within the sponge scaffold.

To overcome this shortcoming, we tested sponges subjected to different overall strain magnitudes. Type X collagen mRNA was quantified and compared from the central regions of the sponges that experienced relatively constant local strains (1/2 of the overall strain). We show that only the center region sample subjected to 7.5% overall strain (3.75% local strain) had a significant increase of type X collagen mRNA level compared to non-loaded control. This result is consistent with the data from the single sponge experiment showing that only local strain more than 2.5% resulted in a significant increase of type X collagen synthesis. This suggests that the threshold of cyclic mechanical induction of type X collagen mRNA production is greater than 2.5% local strain. This *in vitro *observation may have implications for the *in vivo *situations in cartilage. Since type X collagen is a marker of hypertrophic cartilage and osteoarthritic cartilage, our data suggest that mechanical strain above certain threshold (2.5%) may contribute to activation of hypertrophic phenotype during endochondral ossification.

Osteoarthritis has been described as a loss of regulation of chondrocyte maturation, in which chondrocytes are not prevented from progressing from mature chondrocytes to hypertrophic chondrocytes and then through endochondral ossification [12]. Thus, osteoarthritic chondrocytes may share some common properties with embryonic chondrocytes used in this study. Our data suggest that increased local strain beyond a certain threshold in the osteoarthritic lesion may also contribute to the local activation of type X collagen synthesis, similar to its activation in the hypertrophic region. Future studies need to determine whether the threshold of mechanical activation of Col X gene expression is the same between growth plate chondrocytes and the osteoarthritic chondrocytes.

Applied to *in vivo *cartilage function, these results may indicate that certain mechanosensitive gene expression pathways have a threshold for mechanical induction. Differential stress experienced within joint cartilage could be responsible for differential activation of genes involved in matrix remodeling. In support of this hypothesis, application of mechanical stress to normal chondrocytes has revealed that high magnitude cyclic tensile load causes an imbalance between matrix metalloproteinases (MMPs) and tissue inhibitors of matrix metalloproteinases (TIMPs), and an increases of the expression of proinflammatory cytokines IL-1β and TNF-α [14-16]. Thus, differential gene expression activated by local high stress may contribute to osteoarthritic degeneration of some areas of cartilage while other areas remain viable. This may account for heterogeneity of osteoarthritic lesion distribution within a single piece of cartilage or even heterogeneity within osteoarthritic lesions.

Commonly used systems for application of mechanical load to chondrocytes include systems that exert tensile strain, shear stress, hydrostatic pressure, and compressive force [17]. These various forms of mechanical loading differentially up or down regulate cartilage extracellular matrix proteins. For example, studies using cyclic tensile strain have demonstrated an upregulation of several markers of hypertrophic chondrocytes, including type X collagen [2]. Type X collagen up regulation is also found in articular chondrocytes subjected to hydrostatic pressure [18]. Comparison of cyclic tensile strain and hydrostatic pressure found that while both mechanical forces significantly up regulate type X collagen expression, cyclic tension exerts a more pronounced effect on type X collagen up regulation [18]. In addition, examination of the *in vivo *forces exerted on articular cartilage reveals that cyclic tensile strain is analogous to the force created tangential to the joint surface where it articulates and at the cartilage-bone interface where type X collagen is expressed [17]. Thus, cyclic tensile strain is a suitable mechanical loading model for investigation of type X collagen.

Tensile strains applied on a 3D construct in one dimension may lead to compression in the other dimensions. Cyclic compression has also been shown to regulate chondrocyte gene expression [15]. Furthermore, mechanical loading-induced matrix deformation, as measured by the strain of the sponge, leads to a change of the chondrocyte microenvironment within matrix, which includes fluid flow shear stress, streaming potential, hydrostatic pressure, and nutrient transport. All of these factors may contribute to mechanical signaling of chondrocytes [17]. Since our 3D culture system contains these biophysical factors, alteration of the local matrix strain may lead to changes of the microenvironment comprising these factors. It is particularly interesting to link our finding to previous observations [19-21], which suggest that high interstitial fluid flow may be responsible for increased gene expression in local areas. Thus, our data lend support to the idea that altered mechanical microenvironment in cartilage may lead to local activation of gene expression in those areas. Furthermore, the non-uniform strain distributions in Gelfoam sponges, as described in this study, have implications for biomechanical and tissue engineering studies that employ such scaffoldings [2,3,22-26].
